# Tumor infiltrating lymphocyte (TIL) therapy for treating the solid tumors: Challenges and future perspectives

**DOI:** 10.18632/oncotarget.28883

**Published:** 2026-06-08

**Authors:** Bhartendra Sharma, Sukhbir Kaur, Vikas Sharma, Sanjay Soni

**Keywords:** tumor infiltrating lymphocytes, solid tumors, adoptive cellular therapy, immunotherapy

Immunotherapy is one of the effective methods discovered in the treatment of cancer, and adoptive cellular therapy (ACT) represents one of its most promising approaches. Chimeric antigen receptor (CAR) T-cell therapy is the primary form of ACT in which the patients own T-Cells are modified for binding, attacking and eliminating the tumor antigen in hematological malignancies. But CAR T-cell therapy is not effective in treating solid tumors. In this regard, tumor-infiltrating lymphocyte (TIL) therapy has provided good results in clinical trials and benefited patients with different types of solid tumors. TILs are naturally produced mononuclear cells which infiltrate the solid tumor microenvironment (TME). These are also known as the immune cells of the tumor [1].

TIL therapy was discovered by Steven Rosenberg in 1982. He isolated TILs from a mouse tumor and demonstrated that a combination of TILs, cyclophosphamide, and interleukin-2 (IL)-2 was effective in treating hepatic and pulmonary metastatic cancer in mice. Furthermore, in 1988, Steven Rosenberg and his colleagues were the first to administer TIL therapy to humans, reporting favorable clinical outcomes in patients with metastatic melanoma [2, 3].

Generally, there are two types of TILs - intratumoral (iTILs) and stromal (sTILs). iTILs are relatively rare and difficult to detect, whereas sTILs are more frequently present and can be identified more easily. TIL therapy involves isolating the naturally-infiltrating lymphocytes from tumor tissues, *in vitro *expansion and re-infusion of these cells in patients to identify and kill tumor cells. Prior to re-infusion, a non-myeloablative (NMA) lymphodepletion regimen is provided to the patients to suppress the immune system and enhance the effectiveness of infused TIL products. During TIL re-infusion, high dose IL-2 is administered concurrently to promote the survival, proliferation, and anti-tumor activity of the transferred lymphocytes.

TIL therapy has been applied successfully in patients with metastatic melanoma, breast cancer, ovarian cancer, cervical cancer, and other solid tumors, with favorable clinical outcomes reported in multiple studies. However, despite its numerous advantages, TIL therapy has several limitations. For example, the isolation of TILs requires surgical resection of tumor tissue, which can be invasive, risky, and physically stressful for cancer patients. Moreover, even when surgery is feasible, obtaining sufficient tumor tissue for TIL isolation is not always possible. Another critical challenge is overcoming the deficiency of antitumor effector T cells. A highly effective clinical strategy is the combination of specific immunotherapies, such as bispecific molecules (like BiTEs) that physically link T cells to cancer cells, checkpoint inhibitors that block immunosuppressive “brakes”, and topical/intratumoral injections to deliver immune-stimulating drugs directly into the lesion. Furthermore, because of intratumor heterogeneity, it is challenging to develop a universal TIL therapy capable of effectively targeting and eliminating cancer cells across different patients and tumor types.

Another major concern is the diminished capacity of TILs to eradicate tumor cells within the immunosuppressive tumor microenvironment. Overcoming this limitation will require further investigation of T-cell exhaustion markers and the application of advanced single-cell analytical approaches, such as single-cell RNA sequencing, single-cell mass cytometry, and the identification of novel TIL subsets. Additionally, the survival time of infused TIL is short-lived *in vivo*, highlighting the need for further improvements in TIL-therapies.

Therefore, future research should prioritize the development of improved methods for the isolation and expansion of highly tumor-reactive T cells and the investigation of novel combination therapies to maximize clinical benefit [4].

## References

[1] Zhao Y, et al. Cancers (Basel). 2022; 14:4160. 10.3390/cancers14174160. 36077696 PMC9455018

[2] Rosenberg SA, et al. Science. 1986; 233:1318–21. 10.1126/science.3489291. 3489291

[3] Rosenberg SA, et al. N Engl J Med. 1988; 319:1676–80. 10.1056/NEJM198812223192527. 3264384

[4] Kazemi MH, et al. Front Immunol. 2022; 13:1018962. 10.3389/fimmu.2022.1018962. 36389779 PMC9651159

